# Pigment epithelium-derived factor attenuates myocardial fibrosis via inhibiting Endothelial-to-Mesenchymal Transition in rats with acute myocardial infarction

**DOI:** 10.1038/srep41932

**Published:** 2017-02-07

**Authors:** Hao Zhang, Hongliang Hui, Zhimin Li, Jiajun Pan, Xia Jiang, Tengteng Wei, Huazhu Cui, Lei Li, Xulong Yuan, Teng Sun, Zhiwei Liu, Zhongming Zhang, Hongyan Dong

**Affiliations:** 1 Department of Thoracic and Cardiovascular Surgery, Affiliated Hospital of Xuzhou Medical University, Xuzhou 221002, China; 2 Research Facility Center for Morphology, Xuzhou Medical University, Xuzhou 221004, China

## Abstract

Endothelial mesenchymal transition (EndMT) plays a critical role in the pathogenesis and progression of interstitial and perivascular fibrosis after acute myocardial infarction (AMI). Pigment epithelium-derived factor (PEDF) is shown to be a new therapeutic target owing to its protective role in cardiovascular disease. In this study, we tested the hypothesis that PEDF is an endogenous inhibitor of EndMT and represented a novel mechanism for its protective effects against overactive cardiac fibrosis after AMI. Masson’s trichrome (MTC) staining and picrosirius red staining revealed decreased interstitial and perivascular fibrosis in rats overexpressing PEDF. The protective effect of PEDF against EndMT was confirmed by co-labeling of cells with the myofibroblast and endothelial cell markers. In the endothelial cells of microvessels in the ischemic myocardium, the inhibitory effect of PEDF against nuclear translocation of β-catenin was observed through confocal microscopic imaging. The correlation between antifibrotic effect of PEDF and inactivation of β-catenin was confirmed by co-transfecting cells with lentivirus carrying PEDF or PEDF RNAi and plasmids harboring β-catenin siRNA(r) or constitutive activation of mutant β-catenin. Taken together, these results establish a novel finding that PEDF could inhibit EndMT related cardiac fibrosis after AMI by a mechanism dependent on disruption of β-catenin activation and translocation.

Fibrosis in a healing infarct is part of the reparative process, characterized by the fibroblasts accumulation and redundant deposition of extracellular matrix (ECM), and formation of a fibrous scar maintains the integrity of the chamber. Obviously, an overactive fibrotic response, or increased interstitial and perivascular fibrosis in non-infarcted areas would cause a delayed recovery of the ischemic myocardium^1^,^2^. Moreover, overactive cardiac fibrosis would increase ventricular wall stiffness, trigger both systolic and diastolic dysfunction and finally contribute to the conversion from cardiac fibrosis to heart failure^3^. Therefore, preventing aberrant fibrotic reaction after AMI has become a pressing matter.

PEDF, a non-inhibitory member of the serpin family, is widely expressed in diverse human tissues and more prominently in heart tissue^4^. Our previous studies have demonstrated that PEDF shows a variety of biological effects both in the normal heart and infarcted myocardium. Recently, we found that PEDF could improve ischemic heart function and protect cultured H9c2 cells and primary cardiomyocytes against apoptosis and necroptosis under hypoxic condition via the anti-oxidative mechanism^5^,^6^. In fact, an interesting study by Ueda S *et al*. has demonstrated that PEDF inhibits cardiac fibrosis and several relevant factors such as type III collagen^7^. Nevertheless, they have not determined the underlying mechanism of such effects, and the relationship between the endogenous PEDF expression and cardiac fibrosis remains unknown.

Traditionally, adult fibroblasts are considered to be derived directly from embryonic mesenchymal cells^8^,^9^. Recent studies using lineage tracing technique indicate that up to 30% of the fibroblasts formed in the pathogenesis of cardiac fibrosis are derive from endothelial cells via endothelial-to-mesenchymal transition (EndMT)^10^. These disaggregated endothelial cells then start to alter their morphology, migrate to surrounding sites, exhibit endothelial marker depression, and acquire mesenchymal characteristics^2^,^11^,^12^. Investigations that tracked proliferating cell population during cardiac hypertrophy showed that only proliferating fibroblast-like cells were found in the vicinity of the blood vessels^2^,^13^. It is increasingly recognized that during cardiac fibrosis, endothelial cells undergo EndMT and contribute to the total pool of cardiac fibroblasts^10^.

EndMT during embryonic heart development is regulated to a great extent by the TGF-β signaling pathways^14^. Several pieces of evidence have implicated the TGF-β system as a major etiological agent in the pathogenesis of cardiac fibrosis^15^,^16^,^17^. β-Catenin, a central structural component of adhesion complex, also acts as a transcriptional co-activator and play a fundamental role in regulating various biologic processes such as organ development, tissue homeostasis, and pathogenesis of human diseases^18^,^19^. The importance of β-catenin in promoting epithelial-to-mesenchymal transition (EMT) is also demonstrated in many tumor cells with metastatic and invasive capacity^20^,^21^,^22^. It has been shown that TGF-β1 induces nuclear accumulation of β-catenin in tubular cells, and that β-catenin targeting of certain genes results in EMT^23^,^24^. Moreover, TGF-β induction of EMT of endocardial cells is strongly inhibited in mice deficient for endothelial β-catenin^25^.

In this study, we provided evidence of the expression of PEDF linked to diffuse cardiac fibrosis in rats with AMI. By interfering or overexpressing PEDF in myocardium along the infarct border, we aimed to observe the effect of PEDF on interstitial and perivascular fibrosis. And then we explored whether PEDF could ameliorate EndMT-induced cardiac fibrosis through inhibition of β-catenin-signaling pathway.

## Results

### The relationship between cardiac fibrosis and the dynamic expression of PEDF post AMI

**Figure 1:**
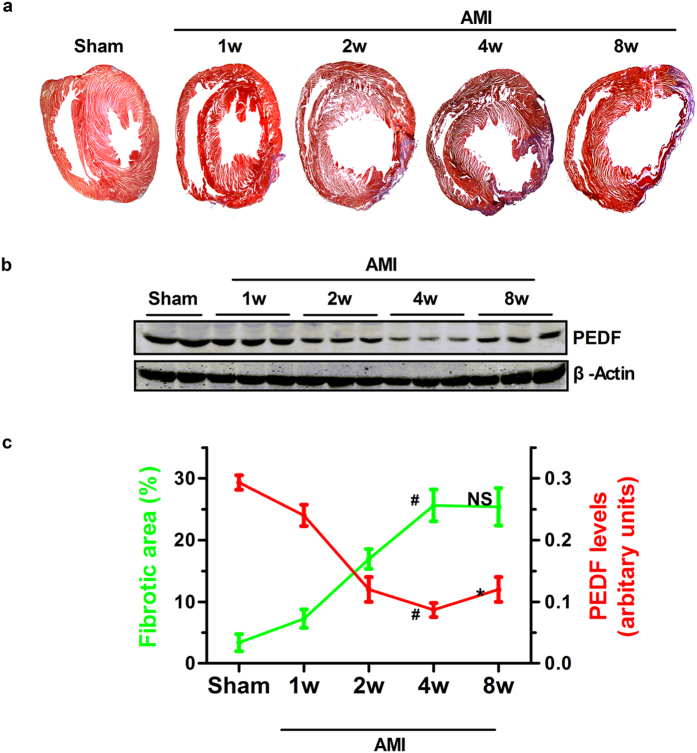
The process of myocardial fibrosis and the dynamic expression of PEDF post AMI. (**a**) Representative MTC staining of the rat heart at 1, 2, 4 and 8 weeks post-AMI. (**b**) Western blot determination of dynamic protein expression of endogenous PEDF in the border zone of the ischemic heart. (**c**) Relationship between cardiac fibrotic size (green) and endogenous PEDF (red). ^#^P < 0.05 vs. the sham group (n = 6); *P < 0.05 vs. the 4w group (n = 6); NS, P > 0.05 vs. 4w group (n = 6).

The fibrotic area and PEDF protein expression were determined in normal and ischemic hearts at 1, 2, 4, or 8 weeks after LAD ligation. MTC staining (Fig. 1a) revealed that the fibrotic area was increased at 1 week and peaked at 4 weeks after LAD ligation as compared with the sham group (P < 0.05) but no significant difference was detected between 4 weeks and 8 weeks after AMI. The results of western blot showed that PEDF protein expression in the border zone were significantly decreased at 1 week and reached a minimum at 4 weeks after AMI compared with the sham group (Fig. 1b and c) (P < 0.05). Our results indicated that there is a reverse pattern between cardiac fibrotic size and PEDF expression at the early phase post AMI.

### PEDF ameliorates cardiac fibrosis and cardiac function in AMI rat heart

**Figure 2:**
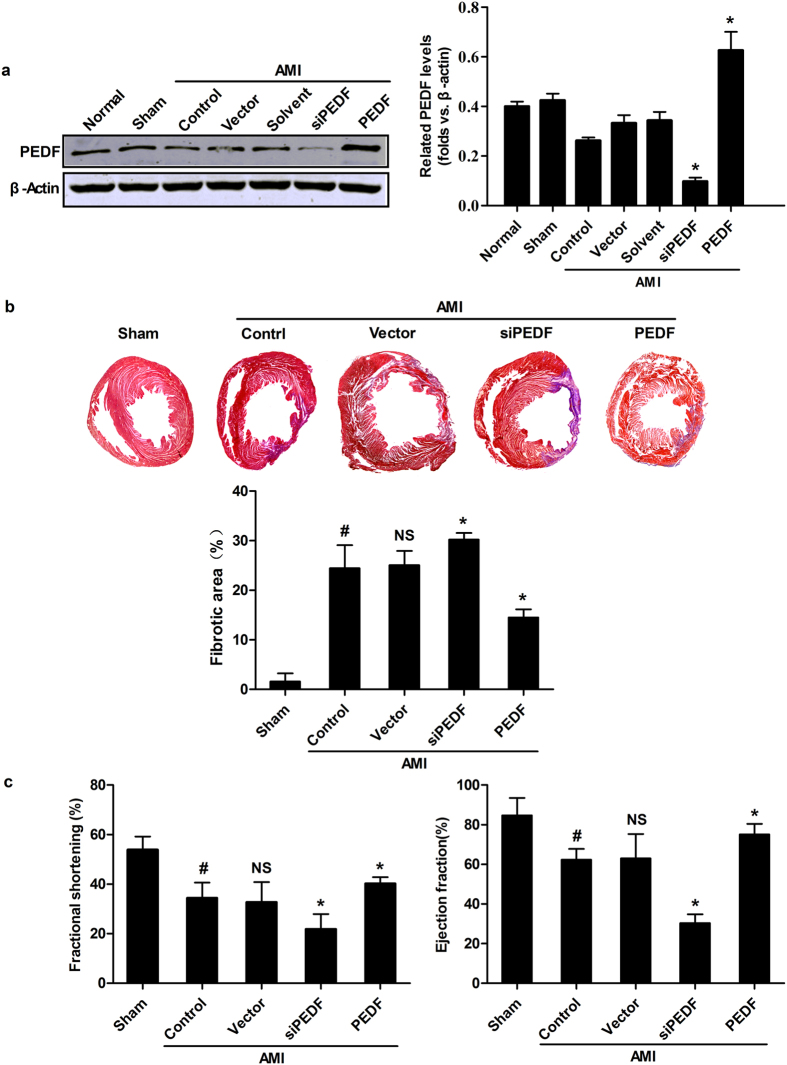
PEDF ameliorated cardiac fibrosis and cardiac function in an AMI rat heart. (**a**) Verification of PEDF expression after intervention with siPEDF or PEDF overexpression at 4 weeks after AMI. Normal, the animal did not undergo surgery; sham control, the animal did not undergo LAD ligation; LAD control, the animal did not undergo any gene transfer after surgery; vector control, LV-CON049 was transferred after surgery; solvent control, 20 μL ENIS was transferred as a solvent control; siPEDF, PEDF-RNAi-lentivirus was transferred after surgery; PEDF, PEDF-lentivirus was transferred after surgery. Values are means ± SD. *P < 0.05 *vs.* the control group (n = 3). (**b**) Representative MTC staining of the heart sections 4 weeks post-AMI and quantitative analysis of fibrotic area. (**c**) Left ventricular fractional shortening (FS) and left ventricular ejection fraction (EF) determination by echocardiography. ^#^P < 0.05 *vs.* the sham group (n = 6); *P < 0.05 *vs.* the vector group (n = 6); NS, P > 0.05 *vs*. the control group (n = 6).

To verify the role of PEDF in ischemic heart, we delivered lentivirus carrying PEDF, PEDF RNAi or LV-CON049 (Vector) by using intramyocardial injections to overexpress or knockdown PEDF in an AMI rat model. The results indicated that PEDF protein expression in the siPEDF group was significantly inhibited compared with the control vector group at 4 weeks after the delivery of the lentivirus (P < 0.05). Whereas in the PEDF group, we found that overexpression of PEDF protein was evident (Fig. 2a).

Based on the results above, we selected the 4-week after AMI as the optimal time point to determine the effect of PEDF on the process of cardiac fibrosis (Fig. 2b) and cardiac dysfunction (Fig. 2c) induced by AMI. In the control group, the fibrotic area was significantly increased compared with the sham group, accompanied by a significant decrease in values of Left ventricular fractional shortening (FS)% and Left ventricular ejection fraction (EF)%, this process was aggravated by knocking-down of PEDF. In contrast, in the PEDF group, the fibrotic area was significantly decreased compared to the vector control group (P < 0.05). Meanwhile, significant improvement in cardiac systolic function was present in the PEDF group compared with the vector control group (P < 0.05). As shown in Fig. 2c, in the vector control group, fractional shortening (FS) and left ventricular ejection fraction (EF) were decreased (~21.2% and 21.7%) compared with the sham group. In the PEDF group, although FS and EF were just increased 7.5% and 12.1% compared with the vector control group, the actual increasing rates of FS and EF were 35.4% (7.5/21.2%) and 55.8% (12.1/21.7%). Moreover, the decreasing rates of FS and EF were 46.1% and 49% in the siPEDF group. Therefore, the intervention with siPEDF or lentiviral PEDF has a certain effect on cardiac function after AMI.

**Figure 3:**
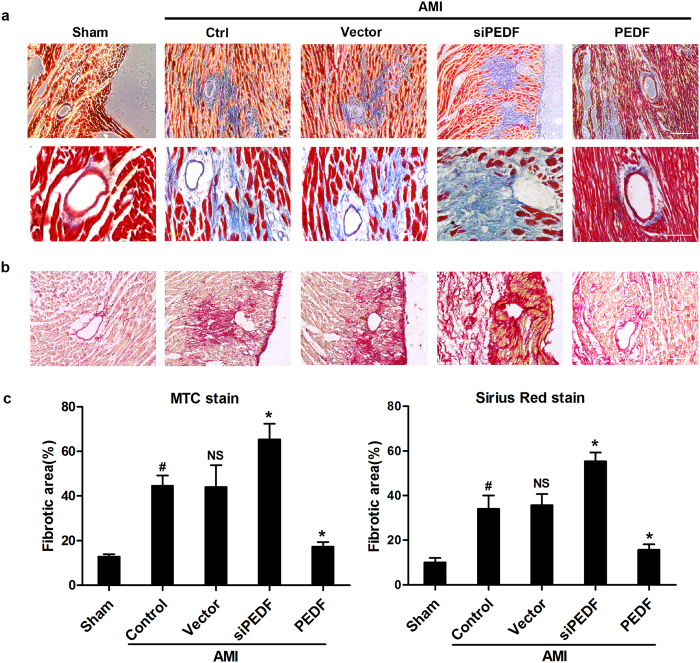
PEDF attenuated cardiac fibrosis around the peri-vascular region in an AMI rat model. (**a**) MTC staining of cardiac fibrosis around the peri-vascular region (Scale bar = 200 μm) and enlarged insets (Scale bar = 60 μm). (**b**) Sirius Red staining of cardiac fibrosis around the peri-vascular region (Scale bar = 200 μm). (**c**) Quantitative analysis of fibrotic area in peri-vascular region. ^#^P < 0.05 *vs.* the sham group (n = 6); *P < 0.05 *vs.* the vector group (n = 6).

MTC staining (Fig. 3a) and Sirius Red staining (Fig. 3b) showed that overexpression of PEDF significantly decreased the fibrotic size and the interstitial collagen expression in the perivascular region. However, knocking-down of PEDF abolished its beneficial effects on cardiac fibrosis (Fig. 3c). Taken together, these results indicated that PEDF attenuated the development of cardiac fibrosis in an AMI rat model, especially around the peri-vascular region.

### PEDF inhibits EndMT *in vivo*

**Figure 4:**
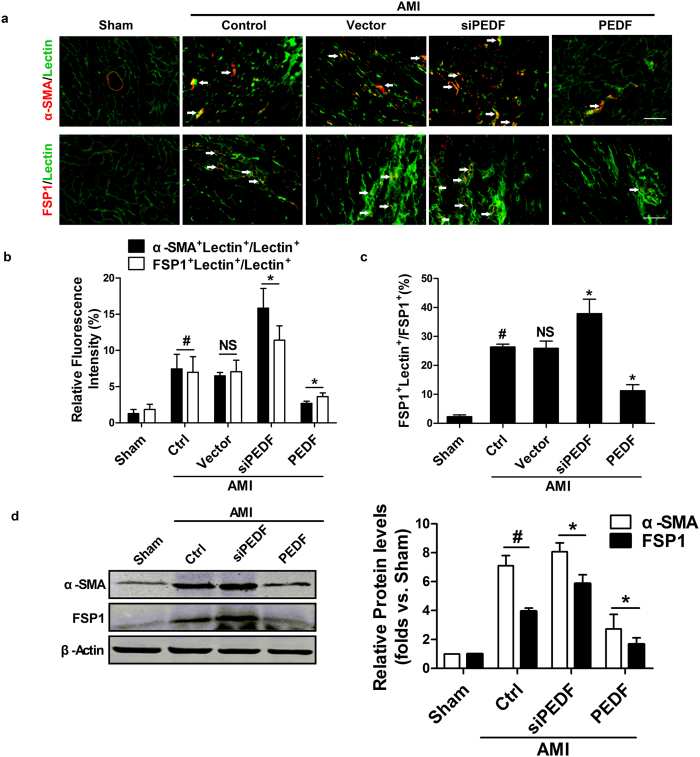
PEDF inhibited EndMT *in vivo*. (**a**) Light microscopy analyses co-expression of α-SMA (red) in lectin-stained endothelial cells (green) in cardiac sections and co-expression of FSP1 (red) in lectin-stained endothelial cells (green) in cardiac sections (Scale bar = 50 μm). White arrows indicate cells co-stained with α-SMA or FSP1 and fluorescein FITC–lectin. (**b**) Quantification of α-SMA^+^ Lectin^+^ cells or FSP1^+^ Lectin^+^ cells versus all Lectin positive cells (the ratio of endothelial cells undergoing EndMT). (**c**) Ratio of FSP1/Lectin double positive cells to all FSP^+^ cells, indicative of the fraction of fibroblasts derived from EndMT. (**d**) Western blot analysis of the expression of α-SMA and FSP1. ^#^P < 0.05 *vs.* the sham group (n = 6); *P < 0.05 *vs.* the vector group (n = 6).

EndMT, one of the critical sources of fibroblast in the fibrotic process, was detected to investigate the exact effect of PEDF on inhibiting cardiac fibrosis. Immunofluorescence determination revealed the co-labeling of cells with the myofibroblast and endothelial cell markers, indicating an early stage of EndMT. We performed double labeling for α-SMA or FSP1 and the endothelial marker Lectin to identify endothelial cells (Lectin+), fibroblasts (α-SMA+ or FSP1+) and cells of endothelial origin that gained of a mesenchymal phenotype (dual α-SMA/Lectin positive cells or dual FSP1/Lectin positive cells)^2^,^26^. We made the quantification of α-SMA+ Lectin+ cells or FSP1+ Lectin+ cells versus all Lectin positive cells to verify the number of endothelial cells undergoing EndMT. Our results showed that the dual α-SMA/Lectin positive cells in the perivascular region were significantly increased in the control groups compared with the sham group (P < 0.05), and this process was aggravated by silencing of PEDF. In contrast, these cells derived via EndMT were significantly reduced in the PEDF group compared with vector control group (P < 0.05) (Fig. 4a and b). Similar results were also found in the immunofluorescence for FSP1/Lectin double positive cells. Moreover, we found that 26.37% of the fibroblasts formed in the pathogenesis of cardiac fibrosis are of endothelial origin. And the ratio of these cells was increased in the siPEDF group but decreased in the PEDF group compared with vector control group (P < 0.05) (Fig. 4c). Meanwhile, western blot analysis showed that PEDF significantly downregulated the expression of α-SMA and FSP1 (Fig. 4d).

### PEDF attenuates β-catenin nuclear translocation *in vivo*

**Figure 5:**
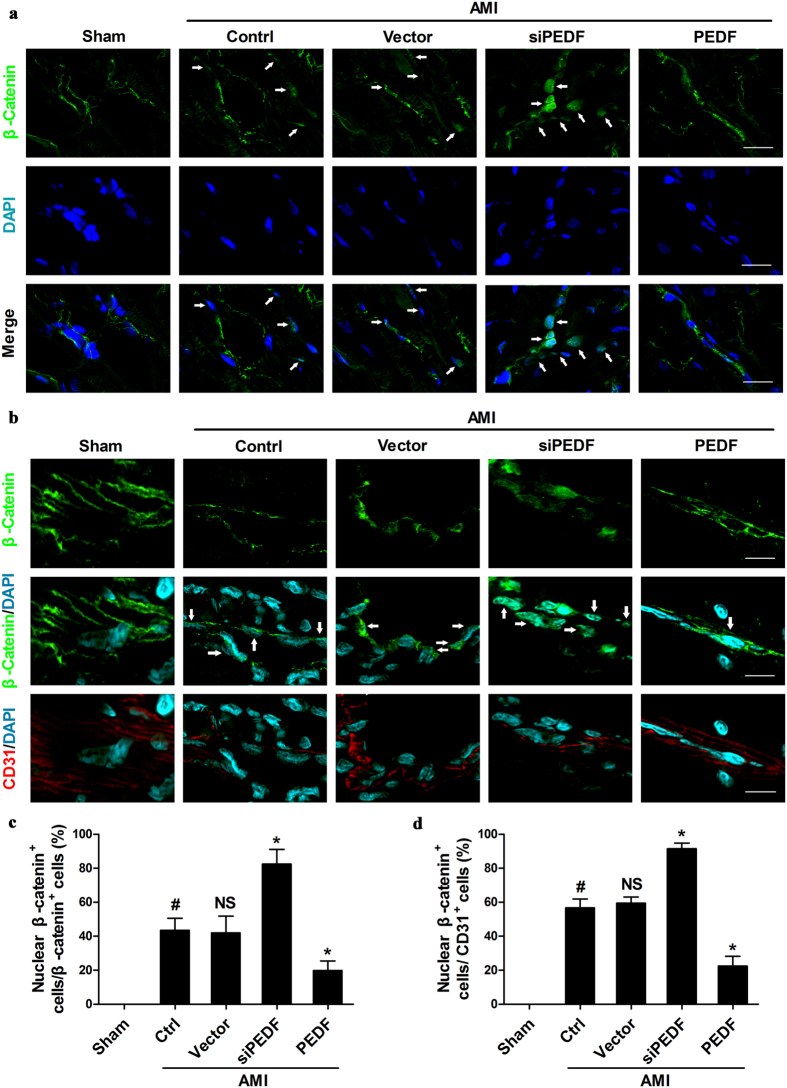
PEDF inhibited β-catenin nuclear translocation *in vivo*. (**a**) Confocal microscopic images of β-catenin immunoreactivities undertaken on hearts under different conditions. β-Catenin (green) staining was performed in each group. Nucleus was stained with DAPI (blue). White arrows indicate nuclear β-catenin staining (Scale bar = 20 μm). (**b**) Confocal double labeling immunofluorescence staining of CD31 (red) and β-catenin (green) undertaken on hearts under different conditions. Nucleus was stained with DAPI (blue). White arrows indicate nuclear β-catenin staining (Scale bar = 20 μm). For the quantitative results shown in (**c**) and (**d**), ^#^P < 0.05 vs. the sham group (n = 5); *P < 0.05 vs. the vector group (n = 5).

We further examined the effect of PEDF on the aberrant nuclear translocation of β-catenin in microvascular endothelial cells post-AMI, which is essentially involved in the progression of EndMT as previous studies have reported. As we know, β-catenin is a main functional protein of endothelial adherens junctions. The results of confocal microscopic images showed that β-catenin located on the membrane of cells in the shame group. After AMI, a nuclear localization of β-catenin was observed, and PEDF siRNA administration increased the number of such cells, whereas the percentage of nuclear β-catenin-accumulating cells was significantly decreased in the PEDF group (Fig. 5a and c). To gather further evidence for the effects of PEDF on nuclear translocation of β-catenin in endothelial cells, double labeling immunofluorescence staining of CD31 and β-catenin was performed (Fig. 5b). We found the nuclear β-catenin was increased in the siPEDF group compared with the vector group, whereas in the PEDF group, the percentage of nuclear β-catenin-accumulating cells was substantially reduced (Fig. 5d). These findings suggested that PEDF inhibited β-catenin nuclear translocation and maintained endothelial junction stability in an AMI rat model.

### PEDF inhibits EndMT induced by TGF-β1 in RCMECs

**Figure 6:**
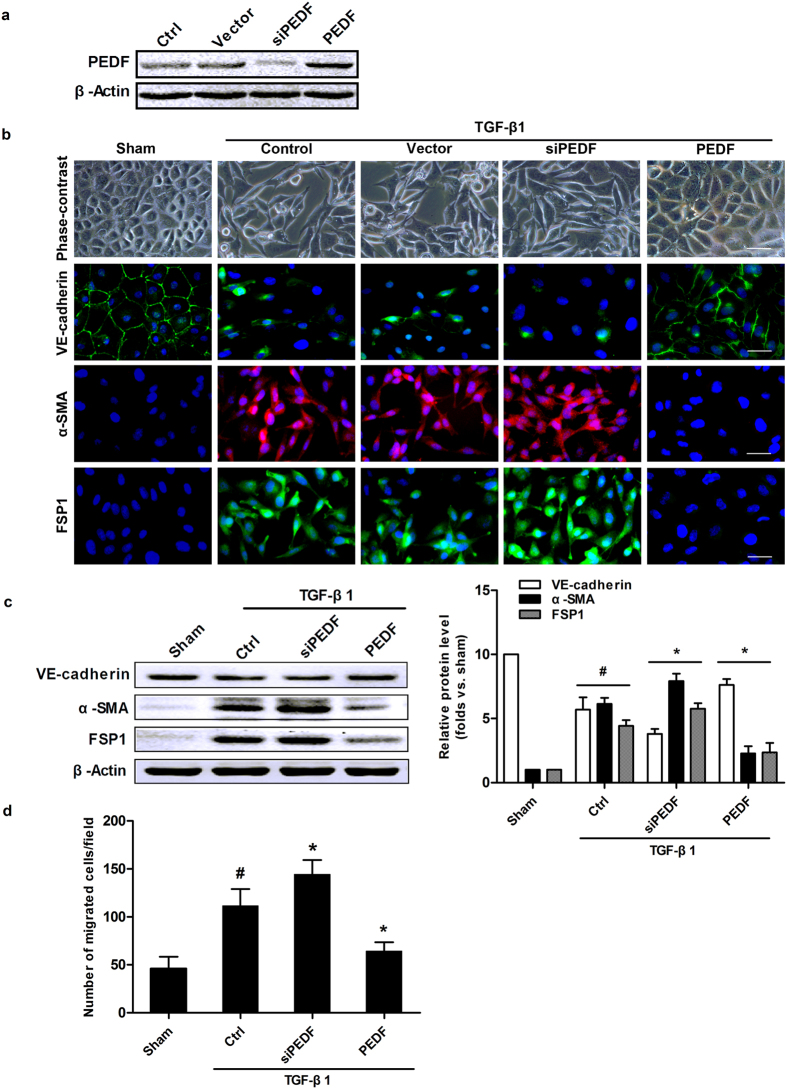
PEDF inhibited TGF-β1-induced EndMT in RCMECs. (**a**) Western blot determination of PEDF protein expression in stable cell lines. (**b**) Phase-contrast images and immunofluorescence images of VE-cadherin, α-SMA, and FSP1 staining in RCMECs untreated (Sham) or treated with 10 ng/mL TGF-β1 for 72 hours (Control), or in stable cell lines with depletion or overexpression of PEDF treated with TGF-β1 for 72 hours (Scale bar = 20 μm). (**c**) Western blot analysis of the expression of VE-cadherin, α-SMA and FSP1. (**d**) Transwell chamber migration assay in cells with corresponding treatments. Migrated cells were microscopically counted after 12-hour incubation. ^#^P < 0.05 *vs.* the sham group (n = 4); *P < 0.05 *vs.* the control group (n = 5).

To further confirm a direct modulation of EndMT by PEDF, we sought to establish stable cell lines overexpressing or silencing PEDF in RCMECs, and cells were exposed to TGF-β1 and evaluated for morphological, phenotypic and functional changes referred to EndMT. As shown in Fig. 6a, PEDF protein expression in ECs was determined by western blot analysis. After culturing in the presence of 10ng/ml TGF-β1 for 72 hours, RCMECs showed a change in morphology from cuboid clustered epithelial cells into spindle-shaped scattered fibroblast-like cells and acquired expression the mesenchymal markers, α-SMA and FSP-1 (Fig. 6b). Immunofluorescence images and Western Blot analysis showed that α-SMA and FSP1 were significantly upregulated in TGF-β1-treated groups compared with the controls, whereas endothelial marker VE-cadherin was downregulated (P < 0.05). Besides, we found that PEDF overexpression could substantially inhibit the expression of α-SMA and FSP1, but silencing of PEDF aggravated the morphological changes and increased the expression of mesenchymal markers in TGF-β1-treated RCMECs (Fig. 6c). During trans-formation, ECs dissociate from the monolayer of tightly cohesive cells at the adluminal surface of the vessel and migrate towards the inner tissue^27^. The migration testing revealed that the migratory capacity of RCMECs was significantly increased after treated with TGF-β1 but this change was effectively attenuated by PEDF overexpression. These observations suggested that PEDF could mitigate the TGF-β1-induced EndMT-associated processes.

### PEDF inhibits EndMT through reducing β-catenin nuclear translocation and transcriptional activity

**Figure 7:**
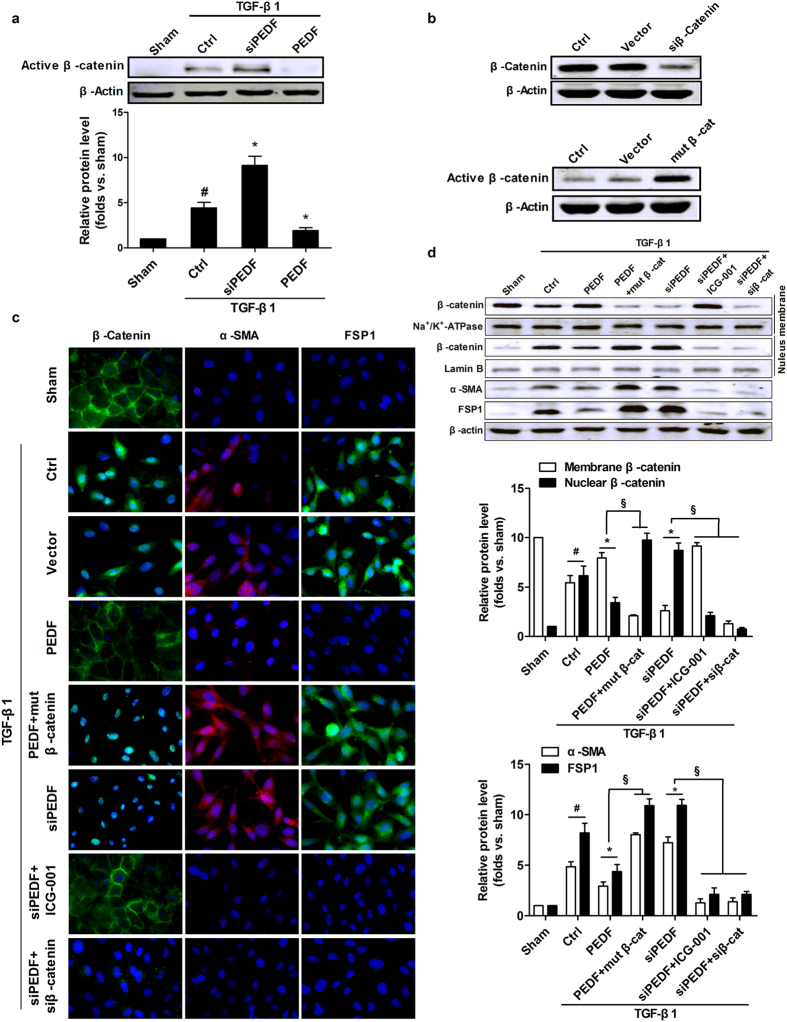
PEDF inhibited TGF-β1-induced EndMT through reducing β-catenin nuclear translocation and transcriptional activity in RCMECs. (**a**) Effect of PEDF on active β-catenin in RCMECs treated with TGF-β1. (**b**) Verification of the functions of plasmids carrying β-catenin siRNA (r) and active mutant of β-catenin in RCMECs. Representative images (**c**) and western blot analysis (**d**) show the localization of β-catenin and the expression of α-SMA and FSP1 in the stable cell lines with differential interference as indicated (Scale bar = 20 μm). The results were quantified by densitometry, membrane and nuclear β-catenin levels were normalized by Na^+^/K^+^-ATPase and Lamin B levels, and α-SMA and FSP1 level were normalized by β-actin. ^#^P < 0.05 vs. the sham group (n = 5); *P < 0.05 vs. the control group (n = 5). ^§^P < 0.05(n = 5).

Given a critical role for β-catenin activation in mediating EndMT^25^, we supposed that inhibition of this signaling might be able to ameliorate the fibroproliferative response of RCMECs to TGF-β1 stimulation. We began by examining whether PEDF would inhibit TGF-β1-induced β-catenin activation in RAECs. Western blot analysis showed that active β-catenin protein level increased in TGF-β1-treated RCMECs, whereas TGF-β1-induced β-catenin activation was significantly abolished with PEDF treatment (Fig. 7a), suggesting that PEDF significantly reduced TGF-β1-induced β-catenin activation.

To directly investigate whether β-catenin transcriptional activity is involved in the inhibition effect of PEDF on EndMT, we co-transfected cells with lentivirus carrying PEDF or PEDF RNAi and plasmids harboring β-catenin siRNA (r) or constitutive activation of mutant β-catenin. The expression of wild-type β-catenin or constitutive activation of mutant β-catenin was confirmed by western blot analysis (Fig. 7b). In TGF-β1-treated RCMECs, we found that β-catenin was translocated from membrane into the nucleus, accompanied by abundant expression of mesenchymal markers. PEDF overexpression effectively degraded TGF-β1-induced β-catenin nuclear translocation and inhibited EndMT. However, knocking down of PEDF increased β-catenin nuclear translocation and transcriptional activity. Furthermore, even though in the presence of excess amounts of PEDF, constitutive activated β-catenin via using mutant β-catenin significantly increased nuclear translocation of β-catenin and up-regulated the level of α-SMA and FSP1 of RCMECs. Moreover, when we utilized β-catenin siRNA or ICG-001, a novel peptidomimetic small molecule which selectively inhibits β-catenin-mediated gene transcription, even under conditions of endogenous PEDF deficiency, TGF-β1-induced EndMT-associated processes was attenuated (Fig. 7c and d). This suggested that the inhibition effect of PEDF on TGF-β1-induced EndMT in RCMECs is mediated predominantly through a β-catenin-dependent pathway.

## Discussion

Recent investigations have suggested that PEDF treatment can potently inhibit the tissue remodeling and cardiac fibrosis in the rat AMI model^7^. However, to date the relationship between endogenous PEDF expression and cardiac fibrosis is still unknown. Meanwhile, whether the effect of PEDF on cardiac fibrosis is relative to inhibiting EndMT and the mechanisms involved also remain to be confirmed. In this study, we reported that endogenous PEDF expression is related to cardiac fibrosis, and PEDF inhibited cardiac fibrosis especially reduced the perivascular cardiac fibrosis in an AMI rat model. We also discovered a new and beneficial role for PEDF which prevented EndMT occurrence and revealed the molecular basis that the role of PEDF in regulation of EndMT via downregulating the transcriptional activation of β-catenin in ischemic heart.

In our study, we evidenced a high expression of endogenous PEDF in the normal myocardium and PEDF expression gradually decreased during AMI, which was consistent with previous study^4^. Additionally, we also detected that cardiac fibrosis developed rapidly following the decreasing expression of PEDF, suggesting that the alteration of PEDF expression may closely relate to fibrotic reaction after AMI. Therefore, we focused our attention on knocking-down or overexpressing PEDF *in vivo* in order to investigate its antifibrotic effects and elucidate possible mechanisms. In this study, we found that PEDF attenuated the development of cardiac fibrosis, presenting a definite anti-fibrotic effect in an AMI rat model. Furthermore, PEDF effectively reduced ECM deposition around the peri-vascular region located in the border of the infarcted area and protected the vascular integrity. Pathophysiologically, a stable number and function of vessels is indispensable for maintaining and restoring cardiac performance in the ischemic heart. Actually, our previous study found that PEDF could improve rat cardiac function by reducing apoptosis, suppressing vascular permeability and limiting infarct size after AMI^6^. However, surviving myocytes would be surrounded by a large number of ECM without intervention, and then the systolic and diastolic function of myocytes would be harmed. These data suggest that PEDF could protect cardiac function from ischemic injury at least by means of reducing cardiac fibrosis, especially inhibiting the peri-vascular fibrosis in the border zone.

As we know, the formation of the excessive ECM is mainly generated by cardiac myofibroblasts^28^. In this study, we found an interesting phenomenon. In the PEDF group, the myocardial fibrotic area was decreased (~10.6%) compared to the vector group (Fig. 2b). However, as shown in MTC staining in Fig. 3, we found that over-express PEDF decreased (~26.8%) fibrotic areas in the perivascular region compared with vector group. Moreover, we found that 26.37% of the fibroblasts formed in the pathogenesis of cardiac fibrosis are derived from endothelial cells in rat AMI model (Fig. 4c). This data suggested that the anti-fibrotic effect after AMI of PEDF is mainly due to its inhibited effect of EndMT around the perivascular region. Thus, in our study, we focused on the effect and the mechanism of PEDF on cardiac fibrosis which is derived from EndMT. Following the alterations of ischemic microenvironment, vascular endothelial cells may undergo EndMT and leave the microvascular bed to interstitium where they appear as myofibroblasts to produce a large amount of ECM deposted in peri-vascular region, leading to malfunction of myocardial microvessels, which in turn reduces the blood supply to the ischemic heart consequently. Thus, EndMT makes it more difficult for myocardial regeneration and revascularization after AMI. Our results *in vivo* showed that PEDF remarkably reduced EndMT and cardiac fibrotic size in peri-vascular region, creating a favorable condition for subsequent recovery of impaired cardiac function.

Amongst the numerous cytokines that regulate epithelial–mesenchymal transition, TGF-β1 has been known to be the most important one^29^. Previous studies demonstrated that endothelial cells of coronary arteries retain the capacity to undergo EndMT on TGF-β treatment^14^. In this study, we stimulated RCMECs by TGF-β1 and demonstrated here that these cells lost specific endothelial markers such as VE-cadherin and expressed mesenchymal or myofibroblastic markers like α-SMA and FSP-1. The enhanced production of ECM could render transformed endothelial cells just as effective as native mesenchymal cells in promoting fibrosis. Moreover, it was found in a migration assay that TGF-β1 stimulation of endothelial cells led to enhanced migratory ability. On the basis of these findings, the potential role of TGF-β1-induced EndMT in RCMECs was confirmed. And the correlation between the inhibitory effects of PEDF on EndMT with its antifibrogenic activity *in vitro* was confirmed by knocking down or overexpressing PEDF. These observations suggested that PEDF served as an endogenous antifibrogenic factor.

Another important finding of this study is that the inhibition effect of PEDF on EndMT is mediated predominantly through a β-catenin dependent pathway. As shown in Fig. 5, nuclear translocation of β-catenin was observed in endothelial cells in an AMI rat model, and this cell population was considerably bigger by knocking-down PEDF. This suggests that lack of PEDF enhances the nuclear β-catenin accumulation. Our results also demonstrated that PEDF significantly attenuated TGF-β1-induced EndMT by inhibiting both the activation and translocation of β-catenin in cultured endothelial cells. Such observations may be important to ensure that β-catenin is an important regulator of the signaling pathway by which PEDF inhibits EndMT. Meanwhile, it is therefore conceivable to speculate the notion that hyperactive β-catenin plays an important role in the pathogenesis of interstitial and perivascular fibrosis. Evidence has been presented that TGF-β1-induced EndMT depends on β-catenin/Smads interaction as transcriptional co-activators but does not depend on canonical Wnt signaling (β-catenin/LEF-1/TCF)^30^,^31^,^32^. This indicated that PEDF could exert beneficial effects on EndMT by suppressing β-catenin/Smad signaling. Intriguingly, previous studies have identified binding of PEDF to LRP6, a Wnt coreceptor, and blocked the canonical Wnt signaling^33^. Therefore, we cannot exclude the possibility that PEDF also could inhibit EndMT via canonical Wnt pathway. There may be some “cross-talking” among β-catenin signaling and other signaling pathways during EndMT process after AMI. Whatever, specific targeting of hyperactive β-catenin signaling with PEDF could represent a novel and effective strategy for therapeutic intervention of cardiac fibrosis, and the detailed molecular mechanism underlying the protective effects against EndMT of PEDF remains to be further investigated.

In conclusions, our study firstly proves that PEDF could inhibit EndMT related cardiac fibrosis after AMI by a mechanism dependent on disruption of β-catenin activation and translocation. These findings are beneficial for further understanding the cardioprotective mechanisms of PEDF and also present a potentiality for establishing a promising strategy for therapy of cardiac fibrosis secondary to AMI.

## Materials and Methods

### Preparations of PEDF Protein

Recombinant rat PEDF (GenBankTM Accession Number NM_177927) was synthesized by Cusabio Biotech, Co., Ltd. (Wuhan, China). In brief, 293T cells were transfected with the recombinant vector pGEX 6P-1 GST-tagged PEDF. GST-tagged PEDF proteins were purified by high pressure liquid chromatography purification (>90% purity) and amino-terminal sequence determination. The resulting proteins were soluble in aqueous solutions.

### Preparation of lentivirus and plasmids

Recombinant lentivirus was prepared as described previously^6^. PEDF over-expression plasmids and the RNAi vector PEDF-RNAi-LV of the PEDF gene that produces PEDF shRNA were successfully constructed and were then successfully packaged by 293T cells. The concentrated titer of virus suspension was 2 × 10^12^ Tu/mL. Plasmid carrying β-catenin siRNA(r) were purchased from Santa Cruz Biotechnology (Santa Cruz, catalog #sc-270011). Plasmid carrying active mutant form of β-catenin (pENTR-N90-beta-catenin) was a gift from Xin Chen (Addgene, catalog #31785)^34^.

### Rat AMI model and intramyocardial gene delivery

Sprague-Dawley (SD) male rats (weighting about 250 ± 10 g, at 8–10 weeks of age, n = 60) were obtained from the Experimental Animal Center of Xuzhou Medical College and housed in a controlled environment. All of the experiments conform to the Guide for the Care and Use of Laboratory Animals published by the US National Institutes of Health (NIH Publication, 8th Edition, 2011). The animal care and experimental protocols were approved by the Xuzhou Medical College Committee on Animal Care. All of the experiments conformed to the international guidelines on the ethical use of animals.

AMI model was established surgically by ligation of the left anterior descending (LAD) coronary artery as described previously^35^. Briefly, the rats were anaesthetized with sodium pentobarbital (60 mg/kg) intraperitoneally. After adequate anesthesia, the animals were intubated with a 14-gauge polyethylene catheter and ventilated with room air using a small animal ventilator (Model683: Harvard Apparatus, Boston, MA, USA). Placed in a supine position, the rat left thoracotomy through the fourth intercostal space was performed under sterile condition, then the pericardium was removed and a 5-0 polypropylene suture (Ethicon, Johnson & Johnson, USA) was placed under the LAD, about 2 mm from the origin. Proximal LAD artery ligation created a reproducibly large lateral wall infarction in the rats. Sham-operated animals underwent an identical surgical procedure without artery ligation. For intramyocardial gene delivery, the rats with AMI were given injection of PEDF-lentivirus or PEDF-RNAi-lentivirus (2 × 10^7^ TU) prepared in 20 μL Enhanced Infection Solution (ENIS, GeneChem, catalog #REVG0002) with a 20-μL syringe and 25-gauge needle along the infarct border immediately after surgery. At 4 weeks after LAD artery ligation, the expression of PEDF in the border zone areas surrounding the infarction was confirmed by western blot analysis (Fig. 2a). Mortality rate within 48 h following AMI surgery was 8%. The animals were maintained on a standard diet after surgery.

### Animal Cardiac Function Evaluation

Echocardiography was conducted under sedation by sodium pentobarbital (30 mg/kg, i.p), as described previously^36^. Two-dimensional-guided (2D) M-mode echocardiography was used to determine left ventricular (LV) chamber volume at systole and diastole and contractile parameters, such as left ventricular end-diastolic dimension (LVEDD), left ventricular end-systolic dimension (LVESD), left ventricular end-diastolic volume (LVEDV) and left ventricular end-systolic volume (LVESV). The left ventricular fractional shortening (LVFS) was calculated as follows: FS (%) = (LVEDD − LVESD)/LVEDD × 100. The ejection fraction (EF) was then derived as EF (%) = (EDV − ESV)/EDV × 100.

### Cell culture and treatment

RCMECs (Cell Biologics, catalog #RN-6024) were used between the third and fifth passage and cultured in endothelial cell medium (ECM, ScienCell, catalog #1001) supplemented with 5% fetal bovine serum (FBS, ScienCell, catalog #0025), 1% endothelial cell growth supplement (ECGS, ScienCell, catalog #1052) and 1% penicillin/streptomycin solution. EndMT induction was performed by RCMECs with 10 ng/mL TGF-β1 (R&D Systems, catalog #240-B)^14^. Medium with TGF-β1 was changed every day. For inhibition of transcriptional activity of β-catenin, cells were typically pretreated with 25 μM ICG-001(Selleckchem, catalog #S2662) at for 0.5 hour^37^, followed by incubation with TGF-β1.

### Establishment of stable cell lines

0.8 × 10^6^ RCMECs were seeded into 60-mm plastic dishes. After the cells reached about 30–35% confluence, PEDF-lentivirus or PEDF-RNAi-lentivirus or LV-CON049 (vector) were transfected following the manufacturer’s protocol at the desired multiplicity of infection (MOI = 20). After 8 hours, transfection medium was removed and fresh medium was added. After an additional 64 hours, GFP co-expression on the construct was used to determine efficiency of viral transduction and the expression of PEDF in the stable cell lines was confirmed by western blot analysis (Fig. 5a).

### Transient gene transfection by siRNA and plasmid DNA

For transient transfection, RCMECs were infected with plasmids (200 ng) carrying β-catenin siRNA or active mutant form of β-catenin or empty plasmid using the Lipofectamine 2000 (Thermo Fisher Scientific, catalog #11668027) reagent according to the instructions specified by the manufacturer. After treatment for 72 h, GFP co-expression on the construct was used to determine efficiency of viral transduction and the expression of wild-type β-catenin or constitutive activation of mutant β-catenin were confirmed by western blot analysis (Fig. 6b).

### Measurement of cardiac fibrosis

Cardiac fibrosis was determined by Masson’s trichrome (MTC) staining (Fuzhou Maixin Biotech Co., Ltd., China). Interstitial collagen was visualized using picrosirius red staining (Vicmed Biotech Co., Ltd., Xuzhou, China)^26^. Isolated perfused hearts were fixed in 10% buffered formalin, embedded in paraffin and sliced into horizontal 5 μm section for MTC staining or Sirius Red staining according to the manufacturer’s protocol. The fibrotic area in each sample was analyzed utilizing microcomputer-assisted Image-Pro Plus. The fibrotic area was determined by calculating the area of positive MTC staining region (blue) or Sirius Red staining region (red) as a proportion of the total LV area or given field area.

### Immunofluorescence analysis

For heart tissue staining, frozen myocardial tissue was horizontally sliced into 5 μm section and mounted on glass slide. Both sections and cells were fixed in 4% paraformaldehyde for 15 min, permeabilized with Triton X-100 (0.1%) and blocked with solution containing 5% bovine serum before applying primary antibody. Specimens were incubated with anti-α-smooth muscle actin (α-SMA) (Abcam, catalog #ab5694; 1:100), anti-fibroblast- specific factor 1 (FSP1) (Abcam, catalog #S100A4; 1:200), anti-VE-cadherin (Abcam, catalog #ab33168; 1:300), anti-CD31(Abcam, catalog #ab24590; 1:200) and anti-β-catenin (Abcam, catalog #ab32572; 1:200) overnight at 4 °C, washed three times in PBS and incubated with the secondary antibody (Life Technologies, catalog #A21207; 1:200) under light-protected conditions for one hour at room temperature and counterstained using 4,6-diamino-2-phenyl indole (DAPI) (KeyGen Biotech, catalog #KGA215–10). Cardiovascular endothelial cells were identified by staining with the lectin BS1-B4 (Sigma, catalog #L-2895) under light-protected conditions at room temperature for 3 hours at and the cover slips were mounted on slides using 50% glycerin. Then, the sections and cells were observed under a fluorescence microscope (Olympus, Tokyo, Japan) or confocal laser scanning microscope (Olympus, Tokyo, Japan).

### Protein extraction

Rat tissue protein was extracted from the myocardium of the peri-infarct zone using a lysis buffer containing 50 mmol/L Tris, 150 mmol/L NaCl, 1% Triton X-100, 0.1% SDS, 1% Na-deoxycholate, 1% protease, (pH 7.5), and complete protease inhibitor cocktail (Sangon Biotech, catalog #C510003). For whole cell lysate, cells were lysed with Cell Total Protein Extraction Kit (Sangon Biotech, catalog #C510003) containing cocktail of phosphatase inhibitors and protease inhibitors. Membrane proteins were extracted using Membrane and Cytoplasmic Protein Extraction Kit (Beyotime, catalog #P0033) and nuclear proteins were extracted using Nuclear and Cytoplasmic Protein Extraction Kit (Beyotime, catalog #P0028) according to the manufacturer’s instructions.

### Western Blotting analysis

Immunoblotting was performed as previously described. In brief, equivalent amount of protein was fractionated on a 10% or 12.5% sodium dodecyl sulfate-polyacrylamide gel electrophoresis (SDS-PAGE) and electro-transferred to 8 μm nitrocellulose membranes (Millipore, catalog #SCWP04700). Nonspecific binding was blocked by incubation with 5% non-fat milk in 0.1% Tween 20/Tris-buffered saline for 30 minutes, followed by overnight incubation at 4 °C with the primary antibody(s). The PEDF antibody (Santa Cruz, catalog #sc-25594) was used at a dilution of 1:500; VE-cadherin at 1:500; FSP1 at 1:500; α-SMA at 1:1000; β-catenin at 1:500; active-β-catenin (Millipore, catalog #05–665) at 1:500; β-actin (Abcam, catalog #ab54724) at 1:1000; Na^+^/K^+^-ATPase (Bioword Technology, catalog #BS14 36); Lamin B (Santa Cruz, catalog #sc-6216). The membranes were washed three times with 0.1% Tween 20/Tris-buffered saline and the appropriate florescence conjugated secondary antibody was added for 1 hour. Washed again, and they were subsequently scanned by Odyssey Infrared Imaging System (Li-Cor Biosciences, USA).

### Migration assays

RCMECs were pretreated with 10 ng/ml TGF-β1 for 3 days before starting the experiment. Migration assays were performed in the 24-well chamber: a polycarbonate membrane (8-μm pore size, Corning Costar, catalog #3422) divided the divided the chamber into upper and lower compartments. A total of 5 × 10^4^ cells in serum free media were seeded into the wells of the upper compartment and 600 μl ECM containing 20% FBS was placed into the wells of the lower compartment of the transwell chamber, which was then incubated at 37 °C in 5% CO_2_ atmosphere. After 12 hours, cells on the upper side of the membrane were taken off with cotton-tipped swabs. The migrated cells on the lower side of the membrane were fixed and stained with 0.4% Trypan Blue and counted with a microscope.

### Statistical analysis

All of the data were expressed as the means ± s.d and were processed with Prism software (version5, GraphPad, La Jolla, CA, USA). One-way analysis of variance followed by Student–Newman–Keuls test was performed for statistical comparisons. Values of P < 0.05 were considered to be statistically significant.

## Additional Information

**How to cite this article**: Zhang, H. *et al*. Pigment epithelium-derived factor attenuates myocardial fibrosis via inhibiting Endothelial-to-Mesenchymal Transition in rats with acute myocardial infarction. *Sci. Rep.* **7**, 41932; doi: 10.1038/srep41932 (2017).

**Publisher's note:** Springer Nature remains neutral with regard to jurisdictional claims in published maps and institutional affiliations.
